# Visfatin Polymorphisms, Lifestyle Risk Factors and Risk of Oral Squamous Cell Carcinoma in a Cohort of Taiwanese Males

**DOI:** 10.7150/ijms.69868

**Published:** 2022-04-11

**Authors:** Kwei-Jing Chen, Ming-Hong Hsieh, Yen-You Lin, Michael Yuan-Chien Chen, Ming-Yu Lien, Shun-Fa Yang, Chih-Hsin Tang

**Affiliations:** 1 School of Dentistry, China Medical University, Taichung 40402, Taiwan.; 2 Department of Dentistry, China Medical University Hospital, Taichung 40447, Taiwan.; 3 School of Medicine, Chung Shan Medical University, Taichung 40201, Taiwan.; 4 Department of Psychiatry, Chung Shan Medical University Hospital, Taichung 40201, Taiwan.; 5 School of Medicine, China Medical University, Taichung 40402, Taiwan.; 6 Division of Hematology and Oncology, Department of Internal Medicine, China Medical University Hospital, Taichung 404332, Taiwan.; 7 Institute of Medicine, Chung Shan Medical University, Taichung 40201, Taiwan.; 8 Department of Medical Research, Chung Shan Medical University Hospital, Taichung 40201, Taiwan.; 9 Graduate Institute of Biomedical Science, China Medical University, Taichung 40402, Taiwan.; 10 Chinese Medicine Research Center, China Medical University, Taichung 40402, Taiwan.; 11 Department of Biotechnology, College of Health Science, Asia University, Taichung 41354, Taiwan.

**Keywords:** genetic polymorphisms, oral squamous cell carcinoma, visfatin

## Abstract

Oral cancer is the eighth greatest generally diagnosed cancer amongst males worldwide and the fourth most generally malignancy amongst Taiwanese males. The pro-inflammatory adipocytokine visfatin promotes tumor growth. Elevated plasma visfatin levels have been identified in patients with oral squamous cell carcinoma (OSCC), although the biological mechanisms underlying the involvement of visfatin in the pathogenesis of OSCC are not well understood. Moreover, no information is available regarding associations between visfatin polymorphisms and carcinogenic lifestyle factors with OSCC. This study, therefore, investigated the effects of four visfatin gene polymorphisms (rs11977021, rs61330082, rs2110385, and rs4730153) and carcinogenic lifestyle factors (betel nut chewing, alcohol consumption and cigarette smoking) on the risk of developing OSCC in 1,275 Taiwanese males with OSCC, and 1,195 healthy males (controls). We also examined the associations between these visfatin genotypes and OSCC histopathological prognostic factors (pathological stage, tumor status, lymph node status, and metastasis). We found that compared with subjects with the CC genotype of SNP rs11977021, those with the CT+TT genotype were less likely to progress OSCC. In addition, an association was found between the rs4730153 variant and lymph node metastasis in the OSCC cohort.

## Introduction

Oral cancer is the eighth most generally diagnosed cancer amongst males worldwide 1 and the fourth most common malignancy amongst Taiwanese males 2. Of all oral cancers, oral squamous cell carcinoma (OSCC) is the greatest general (representing approximately 90% of all malignancies of the oral cavity) 3. Major risk factors for OSCC include human papillomavirus infection 4, 5 and the habitual consumption of carcinogens such as betel nuts, tobacco and alcohol 5. Around 86% of Taiwanese oral tumor patients are habitual betel nut chewers 2. Oral cancer is also associated with genetic aberrations resulting from disturbances in carcinogen metabolism, DNA repair and cell cycle control 6.

The proinflammatory adipocytokine visfatin, also known as nicotinamide phosphoribosyltransferase (NAMPT), is abundantly expressed in human and mouse visceral adipose tissue 7. Increased serum visfatin levels are positively associated with body mass index, cholesterol and insulin resistance index values, and negatively correlated with triglyceride levels in patients with coronary artery calcification 7. Visfatin has also been detected in tumor tissue or plasma from patients with various types of cancers 8, high levels of visfatin in tissue or plasma are associated with human pancreatic ductal adenocarcinoma 9, breast tumor 10, clear cell renal cell carcinoma (ccRCC) 11 and thyroid malignancy 12. And the previous research demonstrated that high levels of visfatin are associated with tumorigenesis through different molecular signaling pathways and genetic polymorphisms 13-15.

Polymorphisms in the visfatin gene are associated with breast cancer and coronary artery disease 7. Visfatin expression affects the regulation of various important factors in development related to tumor progression including tumor survival rate, metastasis and resistance 8. Although elevated plasma visfatin levels have been identified in patients with OSCC 13, the biological mechanisms underlying the involvement of visfatin in the pathogenesis of OSCC are not well understood. Moreover, no information is available regarding associations between visfatin polymorphisms and carcinogenic lifestyle factors with OSCC. This study therefore investigated the effects of visfatin gene polymorphisms and carcinogenic lifestyle factors on the risk of developing OSCC in a cohort of Taiwanese males. We also examined associations between the visfatin genotypes and OSCC histopathological prognostic factors (pathological stage, tumor status, lymph node status, and metastasis).

## Materials and Methods

### Study participants

1,275 male patients in this study were diagnosed with OSCC at Chung Shan Medical University Hospital, Taichung, Taiwan, between January 1, 2010 and December 31, 2019. We randomly selected 1,195 healthy controls without any history of cancer or oral precancerous disease from the Taiwan Biobank, an ongoing prospective study that has been collecting information since 2012 about genes, lifestyle, and environmental risk factors from over 150,000 individuals aged 30-70 years throughout Taiwan. Demographic data and carcinogenic lifestyle behaviors (betel nut chewing, cigarette smoking, alcohol consumption) were recorded. Daily smokers were defined as persons who had smoked at least one cigarette daily in the previous 3 months. Alcohol consumers were defined as persons who consumed more than two alcoholic beverages per day on average. OSCC was graded using the 2018 American Joint Committee on Cancer (AJCC) Cancer Staging Manual (8^th^ edition) 16. A pathologist rated tumor cell differentiation by AJCC classification criteria. All procedures of the study involving human participants satisfied the Declaration of Helsinki criteria. All authors had access to the study data and reviewed and approved this study. Chung Shan Medical University Hospital's Institutional Review Board approved the study investigation (IRB No.CS2-21063). Each participant provided written informed consent before enrolling into the investigation.

### Selection and genotyping of SNPs

Visfatin single-nucleotide polymorphisms (SNPs) rs11977021, rs61330082, rs2110385 and rs4730153 were chosen on the basis of prior research showing that they increased the risk of various cancers 14, 17-19. All SNPs had minor allele frequencies exceeding 5%. Genomic DNA was extracted from 3 mL peripheral blood samples using QIAamp DNA Blood Kits (Qiagen, CA, USA). Allelic discrimination of the SNPs followed previously described assessment procedures 20-22. RNA isolation and RT-qPCR assays followed our previously described procedures 23-25.

### Bioinformatics analysis

The putative functional relevance of visfatin polymorphisms was examined. Data collected from the Genotype-Tissue Expression (GTEx) database were used to recognize relationship between rs4730153 and visfatin performance in esophagus mucosal tissue 26, 27.

### Statistical analysis

Previous research has reported visfatin rs11977021 frequencies for CC, CT and TT genotypes among controls of 23.2%, 54.5% and 22.3%, respectively, and corresponding visfatin rs61330082 frequencies for CC, CT and TT genotypes among controls of 39.7%, 45.1% and 15.2%, respectively 17. Another publication has reported visfatin rs2110385 frequencies for GG, GT and TT genotypes among controls of 75.6%, 24.4% and 0%, respectively (NCBI dbSNP HapMap-HCB population), and visfatin rs4730153 frequencies for GG, GA and AA genotypes among controls of 84.1%, 15.9% and 0%, respectively 28. The frequencies of visfatin polymorphisms in these two studies are comparable with our control samples. We only considered the rs61330082 genotype for calculating the sample size. Given a type I error (α) level of 0.05, type II error (β) level of 0.8, rs61330082 TT genotype among healthy controls of 0.15, odds ratio (OR) of 0.609, the minimum sample size required for cases is 1,195. We therefore recruited 1,195 controls and 1,275 cases for study research.

The differences between the OSCC and control groups were analyzed by the Mann-Whitney *U* test and the Fisher's exact test; between group differences were considered to be statistically significant if p-values were <0.05. Logistic regression was used to calculate odds ratios (ORs) and their 95% confidence intervals (CIs) for associations between genotype frequencies and the risk of OSCC. ORs were adjusted for age, betel quid chewing, cigarette smoking, and alcohol consumption. Clinical staging of OSCC disease was determined in OSCC patients with the variant rs4730153. All collective data were analyzed using Statistical Analytic System (SAS) software version 9.1 for Windows (SAS Institute Inc., CA, USA).

## Results

The study enrolled 1,275 males with OSCC and 1,195 healthy, cancer-free males (controls); all were Taiwanese and living in Taichung, Taiwan. Baseline characteristics of the cases and controls, as well as clinical staging of the OSCC cohort, are shown in Table 1. Approximately half of each study group was aged less than 55 years. The mean age was 53.90 ± 10.02 years in the control group and 55.57 ± 10.76 years in the OSCC group. Compared with controls, higher proportions of patients with OSCC chewed betel nuts, were cigarette smokers and consumed alcohol (*p*<0.001 for all comparisons). Based on the AJCC tumor/node/metastasis (TNM) classification and staging system, 47.0% of patients had clinical stage I/II OSCC and 53.0% had clinical stage III/IV disease; the proportions of patients classified as T1+T2 or T3+T4 status were similar (50.1% and 49.9%, respectively), 65.7% were N0 lymph node status and 34.3% were N1+N2+N3 status, and nearly all patients were metastasis-free (99.2% vs 0.8% with M1 status). OSCC was moderately or poorly differentiated in 86.1% of patients and well-differentiated in 13.9%.

The results of genotyping for the four visfatin SNPs (rs11977021, rs61330082, rs2110385 and rs4730153) in the cases and controls are shown in Table 2. Having the CT+TT genotype of SNP rs11977021 diminish the risk of progressing OSCC, compared with having the CC genotype (adjusted OR [AOR] 0.801; 95% CI, 0.642 to 0.998; *p*=0.048). No significant associations were observed between variants rs61330082, rs2110385 and rs4730153 and OSCC. When visfatin genotypes in cases and controls were evaluated to examine the roles of carcinogenic lifestyle factors on the occurrence of OSCC, we identified that amongst study participants who consumed alcohol, those carrying the AA allele at rs4730153 were significantly less likely than those with the wild-type GG allele to develop OSCC (AOR 0.387; 95% CI, 0.071 to 2.120; *p*=0.046) (Table 3).

An investigation into the association between OSCC clinical status and allelic distributions of visfatin variant rs4730153 revealed no significant associations (Table 4). Next, we found the effect of visfatin gene polymorphisms and carcinogenic lifestyle factors on the clinical status of OSCC. Among the 950 patients who chewed betel nuts, a correlation of rs4730153 variants (GA vs GG) was found for lymph node metastasis (AOR 1.456; 95% CI, 1.022-2.075; *p*=0.038), but no association was found between any of the studied genotypes and clinical stage, tumor size, or OSCC differentiation (Table 5).

**Figure 1:**
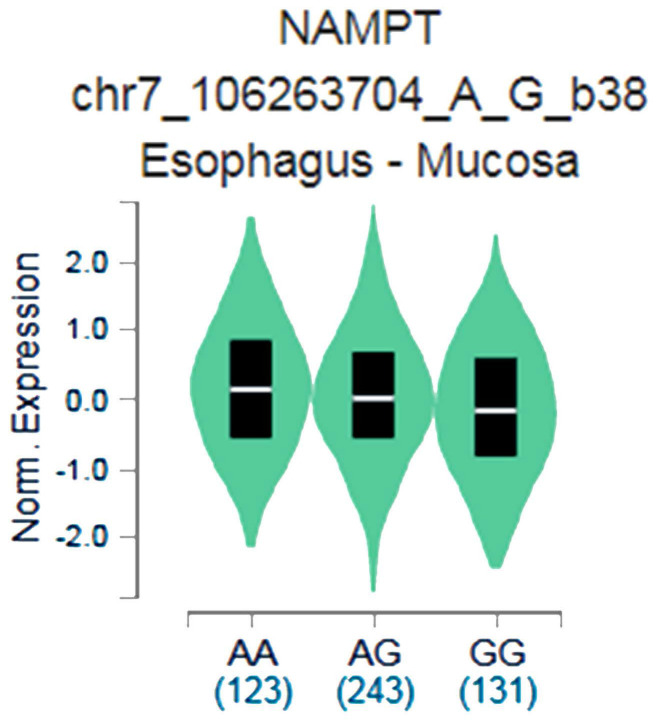
Genotype-Tissue Expression (GTEx) data were used to recognize relevance between visfatin expression and variant rs4730153 in esophagus mucosal tissue.

The GTEx data exhibited that respective carrying the AA allele of variant rs4730153 had significantly higher levels of visfatin compared with patients who had the wild-type GG homozygous genotype (*p*=0.0029; Fig. 1).

## Discussion

OSCC comprises over 90% of oral cancers 29 and although medical progress has witnessed much improvement in the early detection and treatment of OSCC 30, overall 5-year survival is only around 50% 31. Important predictors of outcome include the extent of disease at presentation, whether the patient has clinically palpable lymph nodes and histopathological findings 32. The development of OSCC is influenced by carcinogenic lifestyle behaviors (e.g., betel nut chewing, tobacco smoking and alcohol consumption) 5. According to literature in the Investigation of Nutritional Status and its Clinical Outcomes of Common Cancer (INSCOC) database, research has shown that malnutrition lowers quality of life and increases the risk of infection, the incidence of postoperative complications and the mortality rate 33. The association between nutritional status with treatment outcomes in patients with OSCC should therefore be considered. Our analysis of these factors found that all three potentially facilitate the development of OSCC. This finding is echoed in recent studies that have focused on associations between gene polymorphisms and the occurrence and prognosis of oral cancer 34-36, while carcinogenic lifestyle factors specifically elicit epigenetic changes that increase rates of oral cancers 37, 38.

Visfatin is mostly (not exclusively) secreted by adipose tissue 39 and once released into the extracellular milieu, can behave in a paracrine or endocrine manner in various physiological and pathological conditions, such as cancers 39, 40. High levels of circulating visfatin are reportedly significantly associated with the risk of cancer 41, while higher plasma visfatin levels have been reported in male patients with OSCC, independently of risk factors, which suggests that visfatin is an important contributor to the pathogenesis of OSCC 12. Epidemiological studies have demonstrated higher alcohol consumption increases the risk of OSCC, with strong dose-response relationships 42, 43. The risk of OSCC is three to five times higher among people who have ever consumed alcohol than people who have never consumed alcohol 44, 45. Previous studies have described associations between other SNPs and OSCC risk: for instance, the A>G polymorphism with the rs1412115 variant increased the risk of OSCC in a Chinese Han population 46, while MDM2 SNP 309 and p53 codon 72 polymorphisms influence treatment outcomes of OSCC patients receiving postoperative irradiation 47. In this study, we selected four visfatin gene polymorphisms (rs11977021, rs61330082, rs2110385, and rs4730153) to compare their allelic distributions between healthy, cancer-free subjects and patients with OSCC. We found that OSCC was more likely to develop in subjects who had the CC genotype of SNP rs11977021 compared with those who had the CT+TT genotype (Table 2). In our analysis of alcohol consumption, we observed that OSCC was significantly less likely to develop among study participants carrying the AA allele at rs4730153 compared with those carrying the wild-type GG allele (Table 3). This evidence indicates a critical role for alcohol consumption in association with visfatin polymorphisms and OSCC. Further research on the consequences of SNP variations at the protein level combined with the effect of alcohol consumption may provide insights into new therapeutic targets for OSCC.

Evidence has shown that visfatin promotes the growth of cancers, their metastasis and resistance to cancer therapies 8, 48. Clinically, visfatin levels are associated with metastasis in different types of cancer. Elevated serum visfatin levels are found in patients with small cell lung cancer (SCLC) and brain metastasis 49. In non-SCLC, elevated visfatin plasma levels are correlated with lymph node metastasis 49**.** In colorectal cancer patients, upregulation of circulating serum visfatin is correlated with lymph node metastasis, inflammation and angiogenesis 50. In patients with prostate cancer, visfatin increased the activity and expression of MMP-2 and MMP-9 51. Similar associations between visfatin and lymph node metastasis have been recorded in breast and gastric cancers 52, 53. In this study, we selected visfatin rs4730153 to analyze associations between clinical status and genotypic frequencies in OSCC patients. As shown in Table 4, no differences exist between visfatin rs4730153, various clinical status categories and genotypic frequencies in patients with OSCC. We found that amongst OSCC patients who chewed betel nuts, the GA allele at rs4730153 was significantly associated with lymph node metastasis (Table 5). The result demonstrated that the role of visfatin rs4730153 in OSCC neck lymph node metastasis is probably similar with non-SCLC, prostate, breast and gastric cancers. But, the detail mechanism between the betel nuts chewing, rs4730153 variation and lymph node metastasis should be further investigation.

This study revealed significant associations between alcohol consumption and betel nut chewing and the development of OSCC in Taiwanese males with at least one visfatin polymorphism. The GTEx database has revealed a trend for higher visfatin expression amongst carriers of the AA genotype at rs4730153 compared with carriers of wild-type GG homozygous genotypes, which is mirrored by our SNP data and suggests that the SNP rs4730153 may increase visfatin expression. Limitations of this study need to be addressed. Firstly, information on underlying disease processes is not available from the Taiwan Databank, which meant that the control sample in this study could not be fully investigated regarding disease pathways. Furthermore, the long-term survival rate is also highly affected by the recall rate and will of the patient. More complex investigations using larger study samples and longer follow-up are required to examine associations between visfatin SNPs and OSCC.

In conclusion, this study discusses preliminary results of associations observed between visfatin polymorphisms and carcinogenic lifestyle behaviors in a cohort of Taiwanese males with OSCC. Compared with having the CT+TT genotype, having the CC genotype of SNP rs11977021 increased the risk of developing OSCC. Among the study participants who drank alcohol, the AA allele at rs4730153 was inversely associated with the occurrence of OSCC, suggesting a protective effect. In contrast, among those who chewed betel nuts, the GA allele at rs4730153 was significantly associated with lymph node metastasis.

## Figures and Tables

**Table 1:** The distributions of demographical characteristics in 1,195 controls and 1,275 male patients with OSCC

Variable	Controls (N=1,195)	Patients (N=1,275)	*p* value
**Age (yrs)**			
≤55	607 (50.8%)	640 (50.2%)	*p*=0.766
>55	588 (49.2%)	635 (49.8%)	
**Betel quid chewing**			
No	996 (83.4%)	325 (25.5%)	
Yes	199 (16.6%)	950 (74.5%)	*p* <0.001*
**Daily smoker**			
No	562 (47.0%)	203 (15.9%)	
Yes	633 (53.0%)	1072 (84.1%)	*p* <0.001*
**Alcohol consumer**			
No	961 (80.4%)	674 (52.9%)	
Yes	234 (19.6%)	601 (47.1%)	*p* <0.001*
**Stage**			
I+II		599 (47.0%)	
III+IV		676 (53.0%)	
**Tumor T status**			
T1+T2		639 (50.1%)	
T3+T4		636 (49.9%)	
**Lymph node status**			
N0		838 (65.7%)	
N1+N2+N3		437 (34.3%)	
**Metastasis**			
M0		1265 (99.2%)	
M1		10 (0.8%)	
**Tumor histological grade**			
Well differentiated		177 (13.9%)	
Moderately or poorly differentiated		1098 (86.1%)	

The Mann-Whitney *U* test or Fisher's exact test analyzed differences between healthy controls and patients with oral squamous cell carcinoma. * A *p* value < 0.05 was statistically significant.

**Table 2:** Unadjusted and adjusted odds ratios (ORs) with their 95% confidence intervals (CIs) for OSCC associated with visfatin genotype frequencies

Variable	Controls (N=1,195) (%)	Patients (N=1,275) (%)	OR (95% CI)	AOR (95% CI)^a^
**rs11977021**				
CC	319 (26.7%)	347 (27.2%)	1.000 (reference)	1.000 (reference)
CT	602 (50.4%)	643 (50.4%)	0.982 (0.813-1.185)	0.798 (0.631-1.008)
TT	274 (22.9%)	285 (22.4%)	0.956 (0.764-1.197)	0.807 (0.610-1.068)
CT+TT	876 (73.3%)	928 (72.8%)	0.974 (0.815-1.163)	**0.801(0.642-0.998)^b^**
C allele	1240 (51.9%)	1337 (52.4%)	1.000 (reference)	1.000 (reference)
T allele	1150 (48.1%)	1213 (47.6%)	0.978 (0.875-1.094)	0.894 (0.778-1.028)
**rs61330082**				
GG	313 (26.2%)	339 (26.6%)	1.000 (reference)	1.000 (reference)
GA	602 (50.4%)	644 (50.5%)	0.988 (0.817-1.194)	0.799 (0.632-1.011)
AA	280 (23.4%)	292 (22.9%)	0.963 (0.769-1.205)	0.814 (0.615-1.076)
GA+AA	882 (73.8%)	936 (73.4%)	0.980 (0.819-1.172)	0.804 (0.644-1.004)
G allele	1228 (51.4%)	1322 (51.8%)	1.000 (reference)	1.000 (reference)
A allele	1162 (48.6%)	1228 (48.2%)	0.982 (0.878-1.098)	0.899 (0.782-1.033)
**rs2110385**				
GG	952 (79.7%)	1034 (81.1%)	1.000 (reference)	1.000 (reference)
GT	231 (19.3%)	227 (17.8%)	0.905 (0.738-1.109)	0.990 (0.769-1.274)
TT	12 (1.0%)	14 (1.1%)	1.074 (0.494-2.334)	1.418 (0.566-3.557)
GT+TT	243 (20.3%)	241(18.9%)	0.913 (0.749-1.114)	1.011 (0.791-1.293)
G allele	2135 (89.3%)	2295 (90.0%)	1.000 (reference)	1.000 (reference)
T allele	255 (10.7%)	255 (10.0%)	0.930 (0.774-1.117)	1.031 (0.822-1.293)
**rs4730153**				
GG	958 (80.2%)	1046 (82.1%)	1.000 (reference)	1.000 (reference)
GA	225 (18.8%)	216 (16.9%)	0.879 (0.715-1.081)	0.983 (0.761-1.269)
AA	12 (1.0%)	13 (1.0%)	0.992 (0.451-2.185)	1.389 (0.549-3.513)
GA+AA	237 (19.8%)	229(17.9%)	0.885 (0.723-1.083)	1.003 (0.782-1.287)
G allele	2141 (89.6%)	2308 (90.5%)	1.000 (reference)	1.000 (reference)
A allele	249 (10.4%)	242 (9.5%)	0.902 (0.748-1.086)	1.023 (0.813-1.287)

The ORs with their 95% CIs were estimated by logistic regression models; ^a^ The adjusted odds ratios (AORs) with their 95% CIs were estimated by multiple logistic regression analysis controlling for age, betel quid chewing, cigarette smoking, and alcohol consumption; ^b^ *p* = 0.048.

**Table 3:** Unadjusted and adjusted odds ratios (ORs) with their 95% confidence intervals (CIs) for OSCC associated with visfatin genotype frequencies among alcohol consumers

Variable	Controls (N=234) (%)	Patients (N=601) (%)	OR (95% CI)	AOR (95% CI)^a^
**rs11977021**				
CC	52 (22.2%)	148 (24.6%)	1.000 (reference)	1.000 (reference)
CT	123 (52.6%)	319 (53.1%)	0.911 (0.624-1.330)	0.803 (0.515-1.252)
TT	59 (25.2%)	134 (22.3%)	0.798 (0.514-1.239)	0.662 (0.394-1.110)
CT+TT	182 (77.8%)	453 (75.4%)	0.875 (0.610-1.253)	0.756 (0.496-1.154)
C allele	227 (48.5%)	615 (51.2%)	1.000 (reference)	1.000 (reference)
T allele	241 (51.5%)	587 (48.8%)	0.899 (0.726-1.113)	0.822 (0.640-1.057)
**rs61330082**				
GG	51 (21.8%)	148 (24.6%)	1.000 (reference)	1.000 (reference)
GA	122 (52.1%)	315 (52.4%)	0.890 (0.608-1.302)	0.763 (0.488-1.194)
AA	61 (26.1%)	138 (23.0%)	0.780 (0.503-1.208)	0.652 (0.389-1.093)
GA+AA	183 (78.2%)	453 (75.4%)	0.853 (0.594-1.225)	0.726 (0.474-1.110)
G allele	224 (47.9%)	611 (50.8%)	1.000 (reference)	1.000 (reference)
A allele	244 (52.1%)	591 (49.2%)	0.888 (0.717-1.100)	0.815 (0.634-1.048)
**rs2110385**				
GG	192 (82.1%)	498 (82.9%)	1.000 (reference)	1.000 (reference)
GT	37 (15.8%)	99 (16.5%)	1.032 (0.683-1.559)	1.016 (0.625-1.650)
TT	5 (2.1%)	4 (0.6%)	0.308 (0.082-1.161)	0.468 (0.097-2.258)
GT+TT	42 (17.9%)	103 (17.1%)	0.945 (0.637-1.404)	0.959 (0.601-1.528)
G allele	421 (90.0%)	1095 (91.1%)	1.000 (reference)	1.000 (reference)
T allele	47 (10.0%)	107 (8.9%)	0.875 (0.610-1.256)	0.911 (0.595-1.395)
**rs4730153**				
GG	195 (83.3%)	506 (84.2%)	1.000 (reference)	1.000 (reference)
GA	34 (14.5%)	92 (15.3%)	1.043 (0.681-1.598)	1.015 (0.616-1.674)
AA	5 (2.2%)	3 (0.5%)	**0.231 (0.055-0.977)**	**0.387 (0.071-2.120)**
GA+AA	39 (16.7%)	95 (15.8%)	0.939 (0.624-1.411)	0.947 (0.586-1.531)
G allele	424 (90.6%)	1104 (91.9%)	1.000 (reference)	1.000 (reference)
A allele	44 (9.4%)	98 (8.1%)	0.855 (0.589-1.242)	0.892 (0.574-1.385)

The ORs with their 95% CIs were estimated by logistic regression models; ^a^ Adjusted odds ratios (AORs) with their 95% CIs were estimated by multiple logistic regression analysis controlling for age, betel quid chewing, and cigarette smoking.

**Table 4:** Unadjusted and adjusted odds ratios (ORs) with their 95% confidence intervals (CIs) for various clinical status categories and genotype frequencies of visfatin rs4730153 in males with OSCC (n=1,275)

Variable			OR (95% CI)	AOR (95% CI)
	**Clinical Stage**			
**rs4730153**	**Stage I+II (n=599) (%)**	**Stage III+IV (n=676) (%)**		
GG	496 (82.8%)	550 (81.4%)	1.00	1.00
GA	97 (16.2%)	119 (17.6%)	1.106 (0.824-1.485)	1.101 (0.819-1.479)
AA	6 (1.0%)	7 (1.0%)	1.052 (0.351-3.152)	1.045 (0.347-3.148)
GA+AA	103 (17.2%)	126 (18.6%)	1.103 (0.828-1.470)	1.097 (0.822-1.464)
	**Tumor size**			
**rs4730153**	**≤ T2 (n=639) (%)**	**>T2 (n=636) (%)**		
GG	530 (82.9%)	516 (81.1%)	1.00	1.00
GA	102 (16.0%)	114 (17.9%)	1.148 (0.856-1.539)	1.140 (0.849-1.531)
AA	7 (1.1%)	6 (0.9%)	0.881 (0.294-2.638)	0.836 (0.277-2.521)
GA+AA	109 (17.1%)	120 (18.9%)	1.131 (0.849-1.505)	1.121 (0.841-1.494)
	**Lymph node metastasis**			
**rs4730153**	**No (n=838) (%)**	**Yes (n=437) (%)**		
GG	693 (82.7%)	353 (80.8%)	1.00	1.00
GA	135 (16.1%)	81 (18.5%)	1.178 (0.869-1.596)	1.178 (0.867-1.599)
AA	10 (1.2%)	3 (0.7%)	0.589 (0.161-2.155)	0.599 (0.163-2.203)
GA+AA	145 (17.3%)	84 (19.2%)	1.137 (0.844-1.532)	1.138 (0.844-1.536)
	**Metastasis**			
**rs4730153**	**M0 (n=1265) (%)**	**M1 (n=10) (%)**		
GG	1038 (82.1%)	8 (80.0%)	1.00	1.00
GA	214 (16.9%)	2 (20.0%)	1.213 (0.256-5.750)	1.320 (0.276-6.312)
AA	13 (1.0%)	0 (0.0%)	-	-
GA+AA	227 (17.9%)	2 (20.0%)	1.143 (0.241-5.419)	1.265 (0.264-6.050)
	**Tumor histopathological grade**			
**rs4730153**	**≤ Grade I (n=177) (%)**	**> Grade I (n=1,098) (%)**		
GG	150 (84.8%)	896 (81.6%)	1.00	1.00
GA	25 (14.1%)	191 (17.4%)	1.279 (0.814-2.008)	1.268 (0.806-1.995)
AA	2 (1.1%)	11 (1.0%)	0.921 (0.202-4.195)	0.889 (0.193-4.085)
GA+AA	27 (15.2%)	202 (18.4%)	1.252 (0.809-1.939)	1.240 (0.799-1.924)

Tumor histopathological grade: grade I: well differentiated; grade II: moderately differentiated; grade III: poorly differentiated; The adjusted odds ratios (AORs) and 95% CIs were estimated by multiple logistic regression analysis controlling for age, betel quid chewing, cigarette smoking, and alcohol consumption.

**Table 5:** Unadjusted and adjusted odds ratios (ORs) with their 95% confidence intervals (CIs) for various clinical status categories and genotype frequencies of visfatin rs4730153 in male betel quid chewers with OSCC (n=950)

Variable				
	**Clinical Stage**		**OR (95% CI)**	**AOR (95% CI)**
	**Stage I+II (n=451) (%)**	**Stage III+IV (n=499) (%)**		
**rs4730153**				
GG	381 (84.5%)	403 (80.8%)	1.00	1.00
GA	64 (14.2%)	93 (18.6%)	1.374 (0.970-1.945)	1.361 (0.960-1.930)
AA	6 (1.3%)	3 (0.6%)	0.473 (0.117-1.904)	0.458 (0.113-1.854)
GA+AA	70 (15.5%)	96 (19.2%)	1.297 (0.924-1.819)	1.284 (0.914-1.804)
	**Tumor size**			
**rs4730153**	**≤ T2 (n=484) (%)**	**>T2 (n=466) (%)**		
GG	399 (82.4%)	385 (82.6%)	1.00	1.00
GA	79 (16.3%)	78 (16.7%)	1.023 (0.726-1.442)	1.018 (0.721-1.436)
AA	6 (1.3%)	3 (0.7%)	0.518 (0.129-2.087)	0.490 (0.121-1.984)
GA+AA	85 (17.6%)	81 (17.4%)	0.988 (0.706-1.381)	0.980 (0.700-1.372)
	**Lymph node metastasis**			
**rs4730153**	**No (n=633) (%)**	**Yes (n=317) (%)**		
GG	532 (84.0%)	252 (79.5%)	1.00	1.00
GA	93 (14.7%)	64 (20.2%)	**1.453 (1.022-2.065)^a^**	**1.456 (1.022-2.075)^b^**
AA	8 (1.3%)	1 (0.3%)	0.264 (0.033-2.122)	0.260 (0.032-2.103)
GA+AA	101 (16.0%)	65 (20.5%)	1.359 (0.961-1.920)	1.361 (0.961-1.929)
	**Tumor histopathological grade**			
**rs4730153**	**≤ Grade I (n=144) (%)**	**> Grade I (n=806) (%)**		
GG	121 (84.0%)	663 (82.3%)	1.00	1.00
GA	21 (14.6%)	136 (16.9%)	1.182 (0.718-1.946)	1.177 (0.713-1.941)
AA	2 (1.4%)	7 (0.8%)	0.639 (0.131-3.112)	0.604 (0.123-2.972)
GA+AA	23 (16.0%)	143 (17.7%)	1.135 (0.701-1.836)	1.127 (0.695-1.827)

Tumor histopathological grade: grade I: well differentiated; grade II: moderately differentiated; grade III: poorly differentiated; The adjusted odds ratios (AORs) with their 95% CIs were estimated by multiple logistic regression analysis controlling for age, cigarette smoking, and alcohol consumption; ^a^ *p* = 0.038; ^b^ *p* = 0.038.
